# The Effect of the Number and Identification of Recipients on Organ-Donation Decisions

**DOI:** 10.3389/fpsyg.2021.794422

**Published:** 2021-12-16

**Authors:** Inbal Harel, Tehila Kogut

**Affiliations:** Department of Education & Decision Making and Economic Psychology Center, Ben-Gurion University of the Negev, Be’er Sheva, Israel

**Keywords:** organ donation, willingness to donate, prosocial behavior, identifiable victim effect, scope neglect

## Abstract

We examined how presentations of organ donation cases in the media may affect people’s decisions about organ donation issues. Specifically, we focused on the combined effect of the information about the number of recipients saved by the organs of one deceased person (one vs. four) and the identifiability of the donor and the recipient(s) in organ donation descriptions, on people’s willingness to donate the organs of a deceased relative. Results suggest that reading about more people who were saved by the organs of a deceased donor does not increase willingness to donate. Replicating earlier research, we found that reading about a case of organ donation involving an identified deceased donor, deceased willingness to donate. However, this effect was attenuated when participants read about more recipients who were saved by the donation. Importantly, the presentation that prompted the greatest willingness to donate a deceased relative’s organs was the one that featured an unidentified donor and only one identified recipient. Finally, an explorative investigation into participants’ subconscious thoughts of death following the organ donation story revealed that identifying a deceased organ donor prompts more thoughts of death in the perceiver (regardless of the number of recipients).

## Introduction

“One donor can save eight lives!” This phrase is often used in appeals to members of the public to sign a commitment to donate their organs after death, or to donate the organs of a deceased relative. Moreover, we often encounter—in the printed press, online, or in television reports—of cases of organ donations with information about a deceased donor and about one or several recipients whose lives were saved by that donation.

How might these ads and stories affect readers? In a previous study (Harel et al., 2017), we demonstrated that when participants read about organ donation cases that include identifying information (a name and a photo) about the recipient whose life was saved as a result, it increased their willingness to commit to organ donation themselves, and their willingness to donate (WTD) the organs of a deceased relative. Conversely, identifying the deceased donor was found to induce thoughts of death rather than about saving lives—resulting in fewer participants willing to donate organs (Harel et al., 2017). A study of online news found that in the coverage of organ donation cases in real life, identification of the donor is significantly more common than identification of the recipient—with possibly adverse effects on the incidence of organ donations (Harel et al., 2017).

In the present research, we take one step further in investigating the impact of the presentation of organ donation cases in the media on people’s WTD organs, by examining the role played by the number of recipients saved by the organs of one deceased person, and whether learning about more recipients who were saved as a result reduces thoughts of death, thereby increasing support for organ donation. In addition, we sought to examine the combined effect of the number of recipients saved by the organs of one deceased person and the identifiability of the donor and the recipient[s] in people’s decisions about organ donation issues. Answering these two questions has the potential to make both a theoretical and a practical contribution. First, this investigation will help in understanding the role played by the number of people saved by organ donation, in organ donation decisions (specifically, whether or not *scope neglect* occurs in this context), and to learn about the underlying mechanism (namely, thoughts about death). From the practical standpoint, it will help in identifying the best way to present the issue of organ donations in the media, in a manner that encourages people’s willingness to donate organs.

Research of charitable giving indicates that donation-giving is more likely to be triggered when recipients are identified by name, photograph, or story, than when they are anonymous or merely statistical individuals, even when the identification conveys no meaningful information (Jenni and Loewenstein, 1997; Small and Loewenstein, 2003; Small et al., 2007). When the needs of an identifiable individual are presented, emotional responses (e.g., empathy and compassion) immediately come into play, which increase the incidence of helping. However, when needy individuals are perceived in a negative light—such as when they are perceived responsible for their plight (Kogut, 2011)—identifying information about them may actually increase feelings of anger and blame toward them, reducing willingness to help.

Research on the identified victim effect suggests, however, that identifiability of the recipient increases donations mainly when it involves a single identified individual (Kogut and Ritov, 2005a,b)—and less so when a group of several individuals is presented. As a result, a single identified victim elicits more donations than a group of several victims (whether they are identified or not). Indeed, such is the impact of the number of victims on the willingness to help that it drops dramatically when the number of victims increases even from one to two (Slovic, 2007; Dickert et al., 2015). This *singularity effect*—the preference to help a single identified victim over a group of victims (Kogut and Ritov, 2005a,b) is in line with research of recent decades that consistently shows that people are insensitive to the magnitude of the impact of their support of public causes and of moral decisions (e.g., Desvousges et al., 1993; Kahneman and Ritov, 1994; Baron, 1997; Frederick and Fischhoff, 1998). Hsee and Rottenstreich’s (2004) research suggest that peoples’ subjective values are highly sensitive to the presence or absence of a stimulus (i.e., a change from zero to some number), but they are largely insensitive to further variations in scope, especially when affect-rich stimuli (such as identified victims) are involved. Furthermore, large numbers of victims become dry statistics that fail to spark emotion and feelings, and thus fail to motivate actions (Slovic, 2007). However, it is important to note that some studies have failed to replicate the effect (e.g., Lesner and Rasmussen, 2014; Hart et al., 2018). Moreover, the effect may be restricted to individualistic cultures and societies, and may even reverse in collectivist ones (Kogut et al., 2015; Wang et al., 2015). Furthermore, the effect occurs only in a separate evaluation mode, when prospective donors contemplate helping a single identified recipient or a group of recipients, and are unaware of the alternative condition. In a joint evaluation mode—i.e., when people directly compare the needs of the single individual with those of the group, or when they are asked to choose between them—the decision becomes more rational, and the effect tends to reverse (Kogut and Ritov, 2005b; Wiss et al., 2015; Erlandsson, 2021). Finally, manipulations to increase rational thinking (versus intuitive or emotional thinking modes) and to enhance self-efficacy, attenuated the effect (Small et al., 2007; Sharma and Morwitz, 2016), highlighting the emotional origins of the preference to help single identified individuals.

As previously noted, the presentation of a victim in need of help may be fundamentally different from the presentation of prospective donors and recipients of organ donations. When people donate money to help an identified victim, they believe that their donation will directly help that specific individual—whereas, with organ donations, the commitment to help is directed at an unknown future recipient, and in the unfortunate event of their own death (or that of a close family member). Thus, when a specific case of a prospective organ donation recipient is presented, it can only be by way of illustration, rather than as an actual request for help (Harel et al., 2017).

Moreover, when people consider the issue of organ donations, they are confronted with the disturbing thought of their own demise, or that of a relative. According to terror management theory (e.g., Greenberg et al., 1997), prosocial action helps to suppress anxiety-inducing thoughts of death. Thus, people may act prosocially to shield themselves from the looming prospect of their own mortality—inasmuch that, by helping others, they feel more valuable, and the world seems more meaningful (Jonas et al., 2002). However, Hirschberger et al. (2008) found that, when an appeal for help makes the prospect of one’s own death all the more salient, people may react by setting it aside, and avoiding appeals to help altogether. For example, in one of their studies, mortality-salience manipulation increased charitable donations, but decreased organ donor card registrations (compared with a control condition).

To the best of our knowledge, Harel et al.’s (2017) study is the first to use identified prospective recipients to illustrate an issue (i.e., as individuals who have been saved by organ transplants that had already taken place), rather than as the actual beneficiaries of the decision to donate. In addition, to date, this is the only study that has examined the *identifiability effect* in the context of organ-donation decisions. However, in that study, the recipient was always a single individual, and the donated organ was always a kidney. The research on scope insensitivity and on the singularity effect of identified victims, as reviewed above, raises the question of whether presenting more than one individual who has been saved by organ donations would boost support for organ donations among the public.

This question is important from a theoretical perspective, since while stories about several individuals being saved by the donation of organs of a deceased person may boost organ donations—by prompting thoughts about the lives being saved (rather than about death) (Harel et al., 2017)—they may also reduce WTD due to the natural human tendency to scope insensitivity and the difficulty to adopt the perspective of several other individuals (as opposed to one individual—Slovic, 2007).

In light of recent appeals for organ donations that highlight the fact that one dead person can save the lives of nine people, it is also important to examine this strategy from a practical perspective.

In the present research, we sought to examine the combined impact of the identifiability of the donor and the recipient, and their number (one vs. four recipients) on organ-donation decisions. To this end, we chose to focus on the decision to donate the organs of a deceased close relative (rather than one’s own), since it covers all prospective donors, including those who are willing or have already committed to donate their own organs after death.

In light of the findings of Harel et al. (2017), we expected vivid identifying information about the donor (a deceased individual who has donated his or her organs) to reduce participants’ WTD organs, since such details about deceased donors has been found to prompt thoughts of death (rather than saving lives), decreasing WTD. However, we expected that telling participants that four (rather than one) organ recipients were saved by the donation of organs of a deceased person would attenuate this effect, as it may prompt thoughts about saving lives.

When the deceased donor is left unidentified, we expected identifying information about only one prospective recipient to prompt greater support for organ donations, especially when only one such recipient is presented—in line with the research on the singularity effect, which states that people are more likely to sympathize with, and tend to take the perspective of, a single identified victim, than when a group of such victims with the same need are involved.

To examine these predictions, we used the study design used by Harel et al. (2017), whereby participants read about a recent case of a young man who had been killed in a car accident and whose organs saved the life of another young man. In Study 1, we included eight between-subject conditions in a 2 × 2 × 2 design, varying the identifiability of the donor (identified vs. unidentified), the identifiability of the recipient (identified vs. unidentified), and the number of recipients saved by the organ donation (one vs. four). After reading the story, participants were asked if they were WTD the organs of a deceased family member. In Study 2, we used the same basic description to examine whether reading about more recipients whose lives had been saved by the donation of organs of one deceased donor prompted thoughts of saving lives rather than of death, by examined participants’ subconscious thoughts of death, using a word-completion task.

## Study 1

### Method

To determine the number of participants to recruit for the study, we conducted a power analysis by means of the G*Power computer application (Erdfelder et al., 1996). This indicated that a sample of approximately 300 people would be sufficient to detect a small-to-medium effect size (*f* = 0.15), with a power of 80%. Accordingly, we recruited 304 undergraduate students at Ben-Gurion University (72% female, mean age = 24.39 y, SD = 3.30), through an online subject pool in exchange for monetary prizes—to complete a short survey online. Participants were randomly assigned to one of eight experimental conditions, in a 2 × 2 × 2 design of Donor’s Identification (identified vs. unidentified), Recipient’s Identification (identified vs. unidentified), and the Number of Recipients (*1* vs. *4*), as explained below.

Participants first read a story (adopted from Harel et al., 2017) about a young man who had been killed in a car accident the previous week. He was a registered organ donor, so his parents decided to donate his organs. His kidney [heart, pancreas, two kidneys] was [were] transplanted into the body of another young man [four young men], whose life was [lives were] saved as a result. In the Identified Donor condition, the name and picture of the deceased donor were presented; in the Identified Recipient[s] condition, the same name[s] and picture[s] were attributed to the organ recipient[s]. We used five different typical photos of young men in their twenties to identify the donor and the recipients, while randomly varying the photos in the Identified Donor and the Single Recipient conditions, such that each photo was equally used to identify a single deceased donor and a recipient. In the Four Recipients condition, participants were told that four different organs (from the same deceased donor) were donated to four different recipients: two kidneys, a heart and a pancreas. In the One Recipient condition, we varied the donated organ between-subjects accordingly, such that 1/4 of the participants read about a heart donation, 1/4 about a pancreas donation, and 2/4 about a kidney donation. To enhance involvement, subjects were also asked to indicate whether they had heard about this case (*Yes*/*No*).

Next, participants were asked to imagine that a close relative of theirs had just died, and that the hospital’s medical staff were asking their family to consider donating his organs to save the life of someone waiting for transplantation. Participants were then asked to rate their WTD their deceased relative’s organs on a seven-category scale ranging from *1 (Strongly disagree)* to *7 (Definitely agree)*.

Finally, they were asked to provide demographic information about themselves, including ratings of their degree of religiosity, a variable found in previous studies to be related to willingness to donate organs (1-secualr; 2- traditional; 3- religious; and 4- ultraorthodox) and to indicate whether they themselves were registered organ donors (*Yes*/*No*).

### Results

Willingness to donate the relative’s organs did not significantly differ under the different organ conditions used in the Single Recipient condition (kidney, heart and pancreas; *p* = 0.80), nor under the different photos used to identify the donor and the recipient (*p* = 0.85). We therefore analyzed the Single Recipient condition beyond the different organs and photos.

One hundred and ninety-seven participants reported being registered donors, while 107 were not. Since the participant’s own commitment to organ donations (i.e., whether he/she is a registered donor, or not) was found to play a significant role in the decision about donating the organs of a deceased relative, and may interact with the different presentations (Harel et al., 2017), we used the participant’s consent status (as registered donor or not) as a covariant in the analysis. A 2 × 2 × 2 ANOVA on the WTD the organs of a deceased relative (hereafter, WTD) was conducted, with Donor’s Identification, Recipient’s Identification, and Number of Recipients as predictors.

Results revealed a significant main effect for consent status—such that, as expected, registered donors expressed greater WTD the organs of a deceased relative (*M* = 6.29, SD = 1.05) than unregistered people (*M* = 4.54, SD = 1.62), *F*(1, 295) = 135.93, *p* < 0.001, η_*p*_^2^ = 0.31. No other significant main effects were found.

**FIGURE 1:**
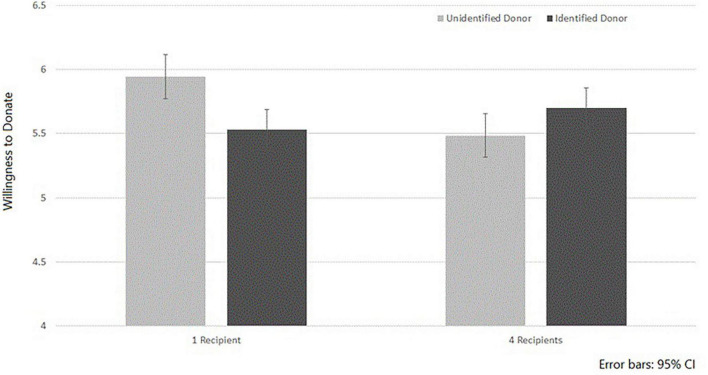
Willingness to donate the organs of a deceased relative as a function of the Donor’s Identification and the Number of Recipients.

The interaction between Donor’s Identification and Number of Recipients was significant *F*(1, 295) = 5.77, *p* = 0.017, η_*p*_^2^ = 0.02. As illustrated in Figure 1, replicating the results of Harel et al. (2017), simple effect analysis shows that when only one recipient was presented, participants who were told about an identified deceased donor (*M* = 5.53, *SD* = 1.67) were less willing to donate the organs of a deceased relative than those whose account talked about an unidentified donor (*M* = 5.94, *SD* = 1.44); *F*(1, 295) = 6.09, *p* = 0.014, η_*p*_^2^ = 0.02. However, when four recipients were saved by the organs of the one deceased donor, identifiability of the donor had no significant effect on willingness to donate—*F*(1, 295) = 0.85, *p* = 0.36, η_*p*_^2^ = 0.003. This suggests that knowing about several people who were saved by the organs of a single dead donor attenuates the effect of Donor’s Identification in reducing support for organ donations. However, reading about four people who were saved by the organ donation did not have a significant effect in increasing WTD.

**FIGURE 2:**
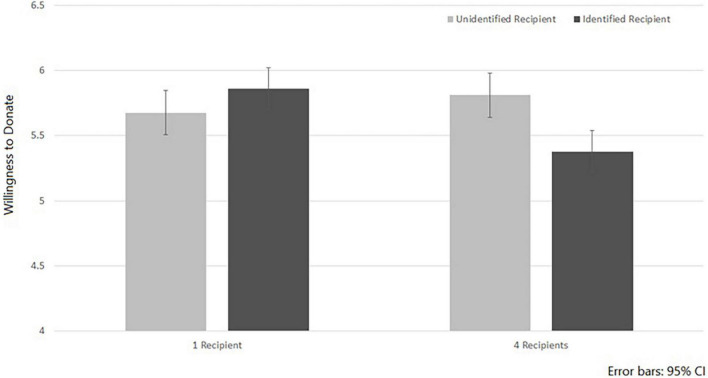
Willingness to donate the organs of a deceased relative as a function of the Recipient’s Identification and the Number of Recipients.

The interaction between Recipient’s Identification and Number of Recipients was also significant *F*(1, 295) = 5.99, *p* = 0.015, η_*p*_^2^ = 0.02. As evident in Figure 2, in the Identified condition, one recipient encouraged greater WTD (M = 5.86, SD = 1.37) than four recipients (M = 5.38, SD = 1.67), *F*(1, 295) = 5.93, *p* = 0.015, η_*p*_^2^ = 0.02; while in the Unidentified condition no significant difference was found between one recipient and four recipients, *F*(1, 295) = 0.96, *p* = 0.33, η_*p*_^2^ = 0.003. This result is in line with previous research on the singularity effect in charitable giving, which suggests that a single identified recipient prompts a greater WTD than a group of recipients. Another way to look at the interaction is to examine the effect of identifiability of a single target and that of a group of four on WTD. A simple effect analysis reveals that identifying four recipients, actually decreased WTD (*M* = 5.38, SD = 1.67), compared to four unidentified recipients (5.89, SD = 1.72), *F*(1, 295) = 7.23, *p* = 0.008, η_*p*_^2^ = 0.024. However, the role of the recipient’s identifiability was far from significance when only one recipient was presented (*F*(1, 295) = 0.47, *p* = 0.49, η_*p*_^2^ = 0.002). This finding is interesting, since it highlights the notion that identifiability may have a negative effect on WTD when several targets are presented (rather than only one). It is possible that providing too much information about several people and various transplanted organs increases stress among the perceivers, distancing them from the situation (e.g., Cameron and Payne, 2011). Alternatively, it might be that thinking about four recipients (rather than one), increased a “calculative mode of thinking” among the participants, which increased their sensitivity to scope (Small et al., 2007; Erlandsson et al., 2016).

**FIGURE 3:**
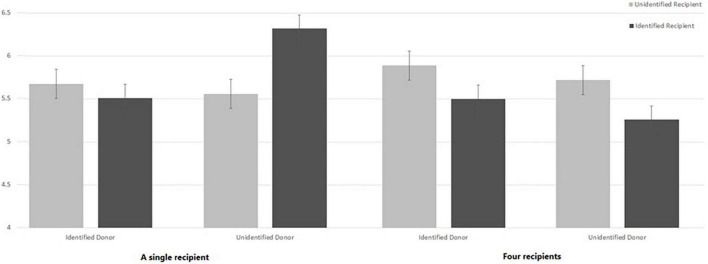
Willingness to donate the organs of a deceased relative (WTD) under the one vs. the four-recipients-conditions, as a function of the Donor’s Identification and recipient(s)’ identifiability.

Finally, the three-way interaction between donor’s identifiability, recipients’ identifiability and the number of recipients approached significance *F*(1, 295) = 3.23, *p* = 0.069, η_*p*_^2^ = 0.011. As illustrated in Figure 3, this interaction suggests that when only one recipient is presented, Donor’s Identification is the only significant predictor for WTD. As found in the study by Harel et al. (2017), when the donor is identified, people are overall less willing to donate the organs of a deceased relative (*M* = 5.54, SD = 1.67) than when the donor is unidentified (*M* = 5.95, SD = 1.44); *F*(1,167) = 4.17, *p* = 0.043, η_*p*_^2^ = 0.024. When four recipients are presented, no significant effects were found, and the main effect of Recipient’s Identification approached significance, suggesting that four unidentified recipients encouraged greater WTD (*M* = 5.80, SD = 1.24) than four identified ones (*M* = 5.38, SD = 1.67) *F*(1, 132) = 2.75, *p* = 0.099, η_*p*_^2^ = 0.02. Previous research on the role of the identifiability of a group of recipients in promoting monetary donations found mixed results: in some studies, it had no effect on donations, while in others it decreased them (Kogut and Ritov, 2005a,b). Replicating the ANOVA with participants’ ratings of their level of religiosity as a covariate revealed similar results. Specifically, both two-way interactions remained significant, while religiosity ratings were not significant (*F*(1, 289) = 2.15, *p* = 0.14).

Judging by Figure 3, the condition that appears to increase WTD (among all eight conditions) is the one in which the deceased donor is not identified, and only one identified recipient is presented. Results of a one-way ANOVA on WTD—with Condition as the independent variable (eight levels), while holding consent-status as a covariant—reveals a significant difference between the eight conditions (*F*(1, 295) = 2.46, *p* = 0.018, η_*p*_^2^ = 0.055. *Post hoc* analysis suggests that participants who were told about an unidentified donor and one identified recipient were significantly more WTD than participants in most of the other conditions, as reported in Table 1. No other significant differences in WTD were found between any other two conditions.

**TABLE 1:** A comparison between WTD under the unidentified donor and an identified recipient condition, and all other conditions.

		Mean difference	SE	Sig
Unidentified donor and 1 identified recipient	Unidentified donor and 1 unidentified recipient	0.57	0.34	0.090
	Identified donor and 4 unidentified recipients	0.43	0.37	0.245
	Identified donor and 1 unidentified recipient	0.76*	0.36	0.039
	Unidentified donor and 4 unidentified recipients	0.60	0.38	0.116
	Identified donor and 1 identified recipient	0.81*	0.36	0.026
	Unidentified donor and 4 identified recipients	1.06*	0.38	0.005
	Identified donor and 4 identified recipients	0.82*	0.38	0.030

*The mean difference is significant at the 0.05 level.

One key finding of Study 1 is that being told about four recipients who were saved by the organs of a single deceased donor attenuates the effect of Donor’s Identification in reducing the willingness to donate. Since previous research (Harel et al., 2017) suggests that the identifiability of the donor is more likely to prompt thoughts of death in people’s minds (as opposed to thoughts about saving lives), resulting in diminished WTD, in Study 2 we sought to explore the degree to which this occurred, and whether being told about more recipients who were saved by the organs of the deceased reduces this tendency.

## Study 2

Study 2 was an exploratory attempt to examine the psychological mechanism that may explain the interaction between identification of the donor and the number of recipients, in terms of the participants’ WTD, as found in Study 1. As noted, previous research suggests that the identifiability of the donor prompts thoughts of death, rather than about saving lives, resulting in diminished WTD. In Study 1, we found that donor identifiability reduced WTD when only one recipient was saved by the organ donation—but when participants were told that *four* recipients were saved by the organs of the deceased, this effect was attenuated, such that their WTD was not significantly different from that of participants who had been told about an unidentified donor.

In Study 2, we examined the salience of death-related thoughts in people’s minds after reading about a case of organ donation. As in Study 1, participants were given a written account about a recent case of a young man who had been killed in a car accident, whose organs were donated to save the lives of others. The study included a 2 × 2 design, manipulating the Donor’s Identification (identified vs. unidentified) and the Number of Recipients (one vs. four) in the story. However, in this case, to keep the design simple, the recipients in all conditions were unidentified. We then examined the participants’ subconscious thoughts of death after the various descriptions, by means of a word-completion task.

### Method

Four hundred and forty undergraduate students at Ben-Gurion University (from a computerized pool of subjects) took part in the study: 63% females, Mage = 26.56, SD = 13.32. Participants were randomly assigned to one of four groups in a 2 × 2 design manipulating the identifiability of the donor (identified vs. unidentified) and the number of organ recipients (one versus four). As in Study 1, participants first read about a young man who had been killed in a car accident, with or without identifying information. They next read that the organ[s] of this man saved the lives of one [four] young men who urgently required them. To examine participants’ subconscious death thoughts, we used a word-completion task involving words that could be completed with either neutral or death-related words. This procedure has been used successfully in previous research to examine people’s accessibility of various subconscious contents (e.g., Greenberg et al., 1994; Arndt et al., 1997; Mikulincer and Florian, 2000; Kogut and Kogut, 2013). The word-completion task included 13 Hebrew word fragments which participants were instructed to complete with the first word that came to their mind by filling in one or two missing letters. Six of the 13 Hebrew word fragments could be completed with neutral or death-related Hebrew words. The death-related words were the Hebrew words for *death*; *funeral*; *grave*; *body*; *deceased*; *mourning*; and “*Shivah*” (a week-long mourning period in *Judaism*). The dependent measure was the number of death-related words with which a participant completed the fragments. Finally, participants provided their demographics including information about whether they are registered donors (yes/no), and religiosity rating (as in Study 1).

In this study we examined accessibility to death-related words after reading about the case of organ donation without assessing WTD, building upon the relationship between thoughts of death and WTD after reading about an identified versus unidentified donor found in previous research (Harel et al., 2017), since several pilot studies (with small samples) revealed that being employed in one of the tasks (completing the connectedness words or making a decision regarding the donation of a deceased relative organs) may distance the participants form the identifiability manipulation, hence weakening its effect on the second task (i.e., only the task that follows the story manipulation is affected by it).

### Results

The number of death-related words completed by the participants in condition is presented in Table 2. Overall, this number ranged between 0–5, *M* = 1.37, *SD* = 1.07. A two-way ANOVA on the number of death-related words by the two independent variables (identifiability and number of recipients) was conducted. Results reveal a significant main effect for Donor’s Identification—*F*(1, 436) = 4.17, *p* = 0.04, η_*p*_^2^ = 0.01—such that reading about an identified deceased donor prompted more thoughts of death among participants (*M* = 1.50, SD = 1.15) than reading the same story with an unidentified donor (*M* = 1.25, SD = 0.95). The Number of Recipients fell far short significance *F*(1, 436) = 0.40, *p* = 0.85, η_*p*_^2^ < 0.001. Although the interaction between identifiability and the Number of Recipients was not significant *F*(1, 436) = 0.82, *p* = 0.37, η*_*p*_^2^* = 0.002, in light of the results of Study 1, we looked at the effect of Donor’s Identification in each of the Recipient Number conditions separately. Simple-effect analysis revealed that Donor’s Identification increased thoughts of death in the One Recipient condition only *F*(1, 436) = 4.32, *p* = 0.038, η*_*p*_^2^* = 0.01, while in the Four Recipients condition Donor’s Identification had no significant impact on thoughts of death, *F*(1, 436) = 0.65, *p* = 0.42, η*_*p*_^2^* = 0.001. Holding Consent Status and level of religiosity constant in the analysis revealed similar results. Specifically, the main effect of Donor’s Identification remained significant (*p* = 0.049) while Consent Status (*p* = 0.56) and Religiosity (*p* = 0.31) did not reveal significant results.

**TABLE 2:** The number of death-related words completed by the participants in condition (Study 2).

Recipients	Identifiability	Mean	SD
One	Unidentified	1.19	0.95
	Identified	1.49	1.21
	Total	1.36	1.11
Four	Unidentified	1.31	0.96
	Identified	1.42	1.09
	Total	1.37	1.03
Total	Unidentified	1.25	0.95
	Identified	1.46	1.15
	Total	1.37	1.07

## Discussion

The results of our investigation of the effect of the presentation of organ donation cases on people’s WTD the organs of a deceased relative, replicated those of previous research by showing that when the participants read about a case of organ donation involving an identified deceased donor, their WTD diminished. However, it also yielded innovative findings about the effect of the number of recipients saved by a single deceased donor on people’s WTD the organs of a deceased relative. As with monetary donation decisions (e.g., Kogut and Ritov, 2005a,b; Slovic, 2007), we found that in the context of organ-donation decisions people are also insensitive to number of victims saved—insofar as reading about more people who were saved by the organs of a deceased donor does not increase WTD. Moreover, when the organ recipients were identified, reading about one person who was saved by organ donation prompted greater WTD than reading about four such individuals. This finding is in line with research that found that people are insensitive to the scope of the problem, especially when emotional triggers are involved (e.g., Hsee and Rottenstreich, 2004; Slovic, 2007). Interestingly, the presentation that prompted the greatest WTD a deceased relative’s organs was the one that featured an unidentified donor and only one identified recipient. This condition combines that of an unidentified donor (which has been found to boost support for organ donation—Harel et al., 2017), and a single identified recipient, which according to research on the identifiable victim effect sparks greater emotions and willingness to help than a group of victims (be they identified or otherwise—Kogut and Ritov, 2005a,b).

Our explorative investigation into participants’ subconscious thoughts of death following the organ donation story replicated previous findings that identifying a deceased organ donor prompts more thoughts of death in the perceiver (Harel et al., 2017). While previous research examined explicit, self-reported thoughts of death, in the present research we used an implicit measure of subconscious death thoughts, as elicited by a word-completion task. In keeping with the pattern found for WTD the organs of a deceased relative in Study 1, we found that identification of the donor significantly increased thoughts of death when only one recipient was saved by the donation, and less so when the participant was told that four people were saved by the donation. Thus, it appears that being told about more people being saved by the organs of a deceased donor actually somewhat weakens the impact of Donor’s Identification on the tendency to think thoughts of death.

In the present research, thoughts of death and WTD were not examined in the same study, since several prior pilot studies (with small samples) showed that only the task that is closely linked to the story (and to the identification manipulation) was influenced by the manipulation—subsequent tasks were not. Future study is therefore needed to further examine the possible role played by thoughts of death in mediating the link between Donor’s Identification and support for organ donation, perhaps by using physiological measures.

Our research has a number of limitations that should be considered when drawing conclusions from it, or when planning related research. First, the experiments were not pre-registered. Specifically, Study 2 was of an explorative nature, and included pilot studies to explore the effect of the order of the two tasks (WTD, and thoughts of death) on the participants’ responses. Thus, future research is needed to replicate these findings, and to examine the mechanisms underpinning the pattern we observed, by means of other methods of gaging thoughts about death. Second, the participants in our experiments are from relatively individualistic societies and cultures. Since the identifiable victim effect has been found mainly in Western cultures (Kogut et al., 2015; Wang et al., 2015), future research is needed to examine how the presentation of organ donations may affect people of more collectivist cultures. Besides its theoretical contributions, our research offers practical implications for efforts to promote organ donations. As suggested by Harel et al. (2017), recruiting people whose lives have been saved by organ donation, identifying them by name, and telling their story may increase media coverage about such individuals, and spur members of the public to think about saving lives when reading about organ donations, and generally to view organ donations in a favorable light. Telling about more people who were saved by the organs of one deceased donor does not seem to be the best strategy to increase support for organ donations. The manipulation we propose to increase willingness to donate organs may be perceived as a way of “programing” people to behave in a certain way. However, the present situation—where only families who have donated the organs of their loved one are telling their story (due to the incentive of commemorating the dead)—appears to be unconsciously affecting the public. Encouraging organ recipients to publish their story may create a more balanced picture of the subject, and increase willingness to donate organs. The greatest positive impact on people’s decisions regarding organ donation, according to the results of our research, appears to be when organ donation reports involve an unidentified deceased donor, and a single identified recipient.

## Data Availability Statement

The raw data supporting the conclusions of this article will be made available by the authors, without undue reservation.

## Ethics Statement

The studies involving human participants were reviewed and approved by Ben-Gurion University of the Negev. The patients/participants provided their written informed consent to participate in this study.

## Author Contributions

IH and TK designed the research, and analyzed the data and wrote the manuscript. IH performed the research. Both authors contributed to the article and approved the submitted version.

## Conflict of Interest

The authors declare that the research was conducted in the absence of any commercial or financial relationships that could be construed as a potential conflict of interest.

## Publisher’s Note

All claims expressed in this article are solely those of the authors and do not necessarily represent those of their affiliated organizations, or those of the publisher, the editors and the reviewers. Any product that may be evaluated in this article, or claim that may be made by its manufacturer, is not guaranteed or endorsed by the publisher.

## References

[B1] Arndt J. Greenberg J. Solomon S. Pyszczynski T. Simon L. (1997). Suppression, accessibility of death-related thoughts, and cultural worldview defense: Exploring the psychodynamics of terror management. *J. Personal. Soc. Psychol.* 73:5. 10.1037//0022-3514.73.1.5 9216076

[B2] Baron J. (1997). Biases in the quantitative measurement of values for public decisions. *Psychol. Bull.* 122 72–88. 10.1037/0033-2909.122.1.72

[B3] Cameron C. D. Payne B. K. (2011). Escaping affect: How motivated emotion regulation creates insensitivity to mass suffering. *J. Personal. Soc. Psychol.* 100 1–15. 10.1037/a0021643 21219076

[B4] Desvousges W. H. Johnson F. R. Dunford R. W. Boyle K. J. Hudson S. P. Wilson K. N. (1993). “Measuring natural resource damages with contingent valuation: Tests of validity and reliability”. In *Contingent valuation: A critical assessment.* ed Hausman J. A. Amsterdam: North-Holland, 91–164. 10.1016/b978-0-444-81469-2.50009-2

[B5] Dickert S. Västfjäll D. Kleber J. Slovic P. (2015). Scope insensitivity: The limits of intuitive valuation of human lives in public policy. *J. Appl. Res. Mem. Cogn.* 4 248–255. 10.1016/j.jarmac.2014.09.002

[B6] Erdfelder E. Faul F. Buchner A. (1996). GPOWER: A general power analysis program. *Behav. Res. Methods Instrum. Comp.* 28 1–11. 10.3758/bf03203630

[B7] Erlandsson A. (2021). Seven (weak and strong) helping effects systematically tested in separate evaluation, joint evaluation and forced choice. *Judgment Dec. Mak.* 16 1113–1154.

[B8] Erlandsson A. Västfjäll D. Sundfelt O. Slovic P. (2016). Argument-inconsistency in charity appeals: Statistical information about the scope of the problem decrease helping toward a single identified victim but not helping toward many non-identified victims in a refugee crisis context. *J. Econ. Psychol.* 56 126–140. 10.1016/j.joep.2016.06.007

[B9] Frederick S. Fischhoff B. (1998). Scope (in)sensitivity in elicited valuations. *Risk Dec. Policy* 3 109–123. 10.1080/135753098348239

[B10] Greenberg J. Pyszczynski T. Solomon S. Simon L. Breus M. (1994). Role of consciousness and accessibility of death-related thoughts in mortality salience effects. *J. Person. Soc. Psychol.* 67:627. 10.1037//0022-3514.67.4.627 7965609

[B11] Greenberg J. Solomon S. Pyszczynski T. (1997). Terror management theory of self-esteem and cultural worldviews: Empirical assessments and conceptual refinements. *Adv. Exp. Soc. Psychol.* 29 61–139. 10.1016/s0065-2601(08)60016-7

[B12] Harel I. Kogut T. Pinchas M. Slovic P. (2017). Effect of media presentations on willingness to commit to organ donation. *Proc. Natl. Acad. Sci.* 114 5159–5164. 10.1073/pnas.1703020114 28461480 PMC5441786

[B13] Hart P. S. Lane D. Chinn S. (2018). The elusive power of the individual victim: Failure to find a difference in the effectiveness of charitable appeals focused on one compared to many victims. *PLoS One* 13:e0199535. 10.1371/journal.pone.0199535 30020998 PMC6051573

[B14] Hirschberger G. Ein-Dor T. Almakias S. (2008). The self-protective altruist: Terror management and the ambivalent nature of prosocial behavior. *Personal. Soc. Psychol. Bull.* 34 666–678. 10.1177/0146167207313933 18303130

[B15] Hsee C. K. Rottenstreich Y. (2004). Music, pandas, and muggers: On the affective psychology of value. *J. Exp. Psychol. Gen.* 133 23–30. 10.1037/0096-3445.133.1.23 14979749

[B16] Jenni K. Loewenstein G. (1997). Explaining the identifiable victim effect. *J. Risk Uncert.* 14 235–257.

[B17] Jonas E. Schimel J. Greenberg J. Pyszczynski T. (2002). The Scrooge effect: Evidence that mortality salience increases prosocial attitudes and behavior. *Personal. Soc. Psychol. Bull.* 28 1342–1353.

[B18] Kahneman D. Ritov I. (1994). Determinants of stated willingness to pay for public goods: A study in the headline method. *J. Risk Uncert.* 9 5–37. 10.1007/BF01073401 32288199 PMC7101763

[B19] Kogut T. (2011). Someone to blame: When identifying a victim decreases helping. *J. Exp. Soc. Psychol.* 47 748–755. 10.1016/j.jesp.2011.02.011

[B20] Kogut T. Kogut E. (2013). Exploring the relationship between adult attachment style and the identifiable victim effect in helping behavior. *J. Exp. Soc. Psychol.* 49 651–660.

[B21] Kogut T. Ritov I. (2005a). The “identified victim” effect: An identified group, or just a single individual? *J. Behav. Dec. Mak.* 18 157–167. 10.1002/bdm.492

[B22] Kogut T. Ritov I. (2005b). The singularity effect of identified victims in separate and joint evaluation. *Org. Behav. Hum. Dec. Proc.* 97 106–116.

[B23] Kogut T. Slovic P. Västfjäll D. (2015). Scope insensitivity in helping decisions: Is it a matter of culture and values? *J. Exp. Psychol. Gen.* 144:1042. 10.1037/a0039708 26372306

[B24] Lesner T. H. Rasmussen O. D. (2014). The identifiable victim effect in charitable giving: evidence from a natural field experiment. *Appl. Econ.* 46 4409–4430.

[B25] Mikulincer M. Florian V. (2000). Exploring individual differences in reactions to mortality salience: Does attachment style regulate terror management mechanisms? *J. Person. Soc. Psychol.* 79:260. 10.1037//0022-3514.79.2.260 10948979

[B26] Sharma E. Morwitz V. G. (2016). Saving the masses: The impact of perceived efficacy on charitable giving to single vs. multiple beneficiaries. *Org. Behav. Hum. Dec. Proc.* 135 45–54.

[B27] Slovic P. (2007). If I look at the mass I will never act: Psychic numbing and genocide. *Judgment Dec. Mak.* 2 1–17.

[B28] Small D. A. Loewenstein G. (2003). The devil you know: The effects of identifiability on punishment. *J. Behav. Dec. Mak.* 18 311–318.

[B29] Small D. A. Loewenstein G. Slovic P. (2007). Sympathy and callousness: The impact of deliberative thought on donations to identifiable and statistical victims. *Org. Behav. Hum. Dec. Proc.* 102 143–153.

[B30] Wang Y. Tang Y. Y. Wang J. (2015). Cultural differences in donation decision-making. *PLoS One* 10:e0138219. 10.1371/journal.pone.0138219 26372014 PMC4570768

[B31] Wiss J. Andersson D. Slovic P. Vastfjall D. Tinghog G. (2015). The influence of identifiability and singularity in moral decision making. *Judgment Dec. Mak.* 10 492–502.

